# Prevalence of Erectile Dysfunction and Associated Factors among Hypertensive Patients Attending Governmental Health Institutions in Gondar City, Northwest Ethiopia: A Cross-Sectional Study

**DOI:** 10.1155/2021/1482500

**Published:** 2021-11-26

**Authors:** Deribew Abebaw Abuhay, Yibeltal Yismaw Gela, Ayechew Adera Getu

**Affiliations:** ^1^Department of Physiology, College of Medicine and Health Sciences, Debre-Tabor University, Debre Tabor, Ethiopia; ^2^Department of Human Physiology, College of Medicine and Health Sciences, University of Gondar, Gondar, Ethiopia

## Abstract

**Introduction:**

Erectile dysfunction is a common sexual problem affecting men with hypertension. It may result in withdrawal from sexual engagement, decreased work productivity, psychosocial problems including poor self-esteem and depression, and reduction in quality of life for both the affected men and their female partners.

**Objective:**

This study was aimed to determine the prevalence of erectile dysfunction and associated factors among hypertensive patients attending governmental health institutions in Gondar city, Northwest Ethiopia.

**Materials and Methods:**

An institutional-based cross-sectional study was conducted on 423 hypertensive men randomly selected using a systematic random sampling technique. Erectile dysfunction was assessed using the International Index of Erectile Function-5 tool. Sociodemographic, clinical, and behavioral factors were also collected using pretested interviewer-administered questionnaires. Data were entered into EpiData version 4.6 and analyzed using Stata-14. Binary logistic regression was performed to identify factors associated with erectile dysfunction. The level of significance was computed at a *p* value ≤ 0.05.

**Results:**

The mean age of the study participants was 58.84 ± 13.52 years. The prevalence of erectile dysfunction among hypertensive men was 46.34% (95% CI: 41.61, 51.12). About 28% of them had a mild form of erectile dysfunction while nearly 6% had severe forms. Age above 60 years (AOR = 3.8, 95% CI: 1.62, 6.55), stage II hypertension (AOR = 3.5, 95% CI: 1.63, 5.74), hypertension duration >10 years (AOR = 2.5, 95% CI:1.12, 4.19), comorbidity (AOR = 1.7, 95% CI: 1.04, 3.15), depression (AOR = 2.35, 95% CI: 1.31, 4.21), and being physically active (AOR = 0.48, 95% CI: 0.28, 0.83) were factors significantly associated with erectile dysfunction.

**Conclusion:**

Nearly half of the study participants had some form of erectile dysfunction, indicating the presence of a high burden of the problem. Assessment of hypertensive men for erectile dysfunction should be part of routine medical care.

## 1. Introduction

Sexual dysfunctions are diminished or absent feelings of sexual interest or desire, absent sexual thoughts or fantasies, and a lack of responsive desire [1]. It is a common problem that affects the quality of life of both patients and their sexual partners [2]. Sexual dysfunction in men is categorized based on the sexual response cycle into erectile dysfunction, hypoactive sexual desire, ejaculation disorders, and orgasmic dysfunctions [3].

Erectile dysfunction (ED) is defined as the consistent or recurrent inability (of a man) to achieve/maintain a penile erection sufficient for satisfactory sexual performance [4]. ED results in withdrawal from sexual intimacy, decreased work productivity, and reduced quality of life [5,6]. It negatively affects employers as men with ED have higher absenteeism rates due to psychosocial reasons that lead to work productivity impairment [7].

The prevalence of ED was different across various parts of the world. ED was found in 66.2% of hypertensive men in Qatar [8], 56.2% of hypertensive patients in Thailand [9], 67% in Israel [10], 61.3% in Bangladesh [11], 35.2% in Greece [12], and 71% in Spain [13]. Moreover, the prevalence of ED was found to be 65.8% in Nigeria [14], 50.6% in Cameroon [15], 43.2% in Egypt [16], and 94.5% in Kenya [17]. Multiple pathogeneses have been identified that link hypertension and ED, including endothelial dysfunction, atherosclerosis, and side effects of antihypertensive medications [18]. Several factors affect erectile function among hypertensive patients, including age, stage, and duration of hypertension, use of antihypertensive medications, depression, and behavioral factors such as excessive alcohol consumption, cigarette smoking, overweight/obesity, and physical inactivity [12,19,20].

Despite the fact that this disorder has been present since ancient times, it is in the past few years that sexual medicine has played an important role in putting together a broad overview of ED [21]. There was no study conducted on ED among hypertensive men in Ethiopia. This study was, therefore, conducted to determine the prevalence of ED and identify associated factors among hypertensive patients attending governmental health institutions in Gondar city.

## 2. Materials and Methods

### 2.1. Study Design, Area, and Period

An Institutional-based cross-sectional study was conducted from April 10 to May 20, 2021, in Gondar city located 727 km away from Addis Ababa, the capital city of Ethiopia. The city is among the ancient and densely populated cities in Ethiopia, having 206,987 people, according to the 2007 Ethiopian Central Statistical Agency office report [22]. There are eight governmental health institutions, namely, University of Gondar Comprehensive Specialized Hospital (UoG CSH), Gondar Health Center, Woleka Health Center, Gebriel Health Center, Maraki Health Center, Mintiwab Health Center, Azezo Health Center, and Teda Health Center that have chronic care units for the diagnosis and treatment of chronic diseases such as hypertension and diabetes mellitus.

### 2.2. Source and Study Populations

#### 2.2.1. Source Population

The source population constitutes all male hypertensive patients on follow-up at chronic care outpatient units of governmental health institutions in Gondar city.

#### 2.2.2. Study Population

Male hypertensive patients were on follow-up at selected governmental health institutions of Gondar city during the period of data collection.

#### 2.2.3. Inclusion Criteria

Male hypertensive patients aged 18 years or above and engaged in sexual activity over the past three months prior to the data collection.

#### 2.2.4. Exclusion Criteria

Male hypertensive patients with a history of pelvic and spinal cord injury, past penile/urethral and prostate surgery, and hearing problems were excluded from the study.

#### 2.2.5. Sample Size Determination

The minimum number of samples required for this study was determined using a single population proportion formula by considering the proportion of ED as 0.5 (since no previous study has been conducted), 95% confidence interval, and 5% marginal error (d):

(1) $$\begin{array}{c} n=\frac{{\left(Z_{\alpha /2}\right)}^{2}P\left(1-P\right)}{d^{2}}, \end{array}$$

where *Z* is statistics at 95% CI = 1.96, *p* is population proportion, and *d* is margin of error:

(2) $$\begin{array}{c} n=\frac{\left(1.962\right)\left(0.5\right)\left(0.5\right)}{0.05}. \end{array}$$

After adding a 10% nonresponse rate, the total sample size was 423.

#### 2.2.6. Sampling Procedure

Both simple and systematic random sampling techniques were employed to select study participants. Among the eight health institutions, four (University of Gondar Comprehensive Specialized Hospital, Gondar Health Center, Maraki Health Center, and Azezo Health Center) were selected using the lottery method. On average, the total number of male hypertensive patients attending the selected health institutions in a month was 480, 160, 120, and 120, respectively. Using the proportional allocation technique, *n*_i_= (n/N)^*∗*^Ni, where *n*_i_ is the number of samples for each study area, *N* = total number of patients, and *N*_i_ = total number of patients in each study area. As a result, 231, 76, 58, and 58 study participants were selected from the University of Gondar Comprehensive Specialized Hospital, Gondar Health Center, Maraki Health Center, and Azezo Health Center, respectively.

Each sample from the study area was selected by using a systematic random sampling technique where the sampling interval (*k*) was obtained by dividing the total number of patients at each health institution by the total sample size. As a result, *K* = 880/423 = 2. Therefore, every other study participant was interviewed, and the first patient to be included in the sample was chosen randomly by the lottery method:

  Study variables.
  Dependent variable.
  Erectile dysfunction (yes/no).
  Independent variables.
  Sociodemographic factors: age, marital status, residence, educational level, occupation, and income.
  Clinical factors: severity of hypertension, duration of hypertension and antihypertension medications, comorbid diseases (diabetes, cardiovascular diseases, chronic kidney disease), depression, overweight.
  Behavioral factors: physical activity, alcohol use, cigarette smoking, khat chewing.
  Operational definitions.
  Hypertension: based on the eighth Joint National Committee guideline (JNC-8), SBP/DBP of 140/90–159/99 and ≥ 160/100 mmHg for all male hypertensive patients with antihypertensive medications was stage I and II hypertension, respectively, while SBP/DBP of <140/90 mmHg was regarded as controlled [23].
  Erectile dysfunction: hypertensive patients who scored 6–25 out of 30 points were reported as having ED according to the International Index of Erectile Function (IIEF-5) [24].
  group of disorders of the heart and blood vessels including coronary heart disease, cerebrovascular disease, peripheral arterial disease, congenital heart disease, rheumatic heart disease, deep vein thrombosis and pulmonary embolism [25].
  Body Mass Index (BMI): hypertensive patients were underweight for BMI<18.5 kg/m^2^ and normal for those with BMI ranging from 18.5 to 24.9 kg/m^2^. Study Cardiovascular diseases: participants were considered overweight for BMI ≥25 kg/m^2^ [26].
  Depression: patients who scored ≥10 out of 27 points from the Patient Health Questionnaire-9 (PHQ-9) were considered as having depression while a score of 0–9 was classified as having no depression [27].
  Physical activity: hypertensive man was considered physically active when engaged in moderate-intensity physical activity for at least 150 minutes per week or in vigorous-intensity physical activity for at least 75 minutes per week, or an equivalent combination of moderate and vigorous-intensity activity [28].
  Ever alcohol drinker: hypertensive patient who used at least 12 drinks his lifetime [29].
  Current alcohol drinkers: hypertensive patients had 3 or more standard drinks per week in the last 30 days [29].
  Former cigarette smokers: a person who has smoked at least 100 cigarettes in his lifetime but who had quit smoking during the time of interview [30].
  Current smoker: a person who has smoked 100 cigarettes in his lifetime and smokes cigarettes within the last 30 days [30].
  Ever khat chewer: study participant who chewed khat at least once in his lifetime [31].

### 2.3. Data Collection Tools and Procedures

Two clinical psychologists and three BSc nurses were involved in data collection with close supervision by the investigator. The presence and severity of ED were measured using an interviewer-administered International Index of Erectile Function (IIEF-5) tool; a brief, reliable, and valid questionnaire found useful in the clinical assessment of sexual activities and treatment outcomes in clinical trials. It is composed of six questions (items) answered on five Likert scales summed up to give a total score of 30. Individuals who scored 6–25 out of 30 points were reported as having ED while IIEF-5 scores of 26–30 were reported as not having ED. The severity of ED was classified as mild (17–25 points), moderate (11–16 points), and severe (6–10 points) [32].

The weight of the study participants was measured on light cloth and bare feet and recorded in kilograms. Height was measured using a stadiometer to the nearest 0.5 centimeters. The weight measuring scale was checked and adjusted at zero level between each measurement. BMI of study participants was computed by dividing their weight in kilograms by the square of their height in meters and interpreted as underweight (<18.5 kg/m^2^), normal (18.5–24.9 kg/m^2^), and overweight (≥25 kg/m^2^) [26].

Blood pressure was measured twice in a sitting position using a standard Mercury sphygmomanometer BP cuff (Mercurial, India) with the appropriate cuff size covering two-thirds of the upper arm after the participant rested for at least five minutes and no smoking or drinking caffeinated beverages for 30 minutes before measurement. The average of two readings taken five minutes apart was recorded as the blood pressure. The staging of hypertension was categorized according to the JNC-8 guideline.

The time since hypertension was diagnosed and started taking antihypertensive medications and the presence of comorbidities were taken from the patient's medical records. Depression status was measured using the PHQ-9 tool designed to evaluate the presence and severity of depressive symptoms over the last two weeks preceding the study. According to this tool, a PHQ-9 score of 0–9 was considered as having no depression while a score of 10 and above was taken as having depression (27).

Physical activity was assessed using the global physical activity questionnaire recommended by WHO [33]. Other self-reported behavioral data (alcohol consumption, cigarette smoking, and khat chewing habits) were collected using a standardized questionnaire adopted from World Health Organization (WHO) STEPS surveillance manual [31]. To prevent the spread of COVID-19 during the data collection period, study participants and data collectors applied COVID-19 prevention measures such as wearing face masks and using hand sanitizers.

### 2.4. Data Quality Control and Management

Clinical psychologists and BSC nurses who work in the chronic care units were used as data collectors to decrease nonresponse rates and increase the reliability of results. The questionnaire was pretested on 5% [20] of male hypertensive patients attending their follow-up at Ibex primary hospital, and modifications were made on the basis of the findings. Training and practical demonstrations on interview techniques and measurement procedures were given to data collectors for one day. English version of the questionnaire was translated to Amharic version for consistency. Completeness of the information from questionnaires was checked every day after data collection. Confidentiality of the information was assured from the data collectors and principal investigator side.

### 2.5. Data Analysis

The collected data were coded and entered into EpiData version 4.6 and then exported to Stata-14 for analysis. Descriptive statistics such as frequency, percentage, and other summary statistics were used for describing sociodemographic variables and determining the prevalence of ED. Tables, figures, and texts were used to present the findings. Bivariable logistic regression analysis was applied to identify factors associated with ED. All variables with *p* value ≤ 0.2 from bivariable logistic regression analysis were entered into the multivariable logistic regression model. The strength of association between dependent and associated factors was determined using the odds ratio with a 95% confidence interval. For all analyses, the statistically significant level was fixed at a *p* value < 0.05.

### 2.6. Ethical Consideration

Ethical approval was obtained from the ethical review committee of the School of Medicine, College of Medicine and Health Sciences, University of Gondar, Ethiopia (Ref. No-433/2021). Permission was obtained from the University of Gondar Comprehensive and Specialized Hospital and the other three health centers. Respondents were informed about the purpose and benefits of the study and gave written consent.

## 3. Results

### 3.1. Sociodemographic Characteristics of Study Participants

A total of 423 male hypertensive patients were involved in the study with a 100% response rate. The mean age of the study participants was 58.84 (±13.52) years, ranging from 26 to 90 years. The majority of the study participants (79.67%) were married (Table 1).

### 3.2. Clinical, Anthropometric, and Behavioral Characteristics of Hypertensive Patients

Of the total respondents, 217 (51.30%) and 66 (15.60%) patients had stages I and II of hypertension, respectively. The median duration of hypertension was four years with IQR from 3 months to 16 years. About 16% of the study participants lived with hypertension for more than 10 years. Among hypertensive patients, 52.95% were on polytherapy. A significant proportion of hypertensive patients (34.28%) had coexisting chronic diseases. The most common coexisting disease was diabetes mellitus, which accounted for 41.4%, followed by cardiovascular disease (29.7%). In our study, we found 34.99% prevalence of depression as presented in Table 2.

### 3.3. Prevalence of Erectile Dysfunction among Hypertensive Patients

**Figure 1:**
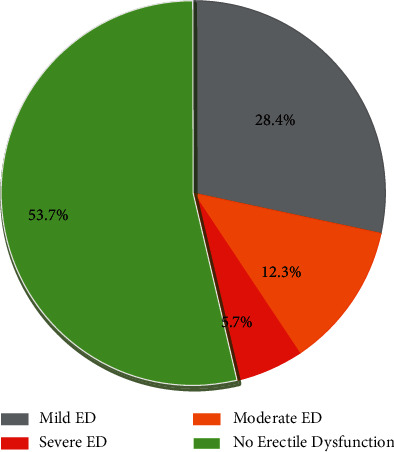
Prevalence of erectile dysfunction among hypertensive patients attending governmental health institutions in Gondar city, Northwest Ethiopia, 2021.

Based on IIEF-5 scores, the overall prevalence of ED was 46.34% (95% CI: 41.6–51.1). Mild ED was found among 120 (28.37%, 95% CI: 24.26–32.87), moderate from 52 (12.29%, 95% CI: 9.48–15.79), and severe form of ED among 24 (5.68%, 95% CI: 3.83–8.34) study participants (Figure 1).

**Figure 2:**
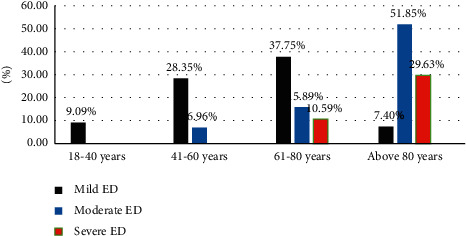
Prevalence and severity of erectile dysfunction based on age group of study participants attending governmental health institutions in Gondar city, Northwest Ethiopia, 2021 (*n* = 423).

Severe and moderate ED was found among 29.6% and 51.85% hypertensive men above 80 years. Similarly, 10.6% and 15.9% hypertensive men aged 61–80 had severe and moderate ED, respectively. Severe ED was not found among those below 61 years (Figure 2).

### 3.4. Factors Associated with Erectile Dysfunction among Hypertensive Patients

In the bivariable logistic regression analysis, independent variables with *p* value of less than 0.2 were passed to be included in the multivariable logistic regression analysis. Accordingly, age, severity of hypertension, duration of hypertension, polytherapy, presence of comorbidities, depression, and physical activity were statistically associated with ED.

Age above 80 years (AOR = 5.29, 95% CI: 3.38–8.07) and 61–80 years (AOR = 3.86, 95% CI: 1.62–6.55), stage I hypertension (AOR = 2.79, 95% CI: 1.51–5.09) and stage II hypertension (AOR = 3.54, 95% CI: 1.63–5.74), duration of hypertension for more than 10 years (AOR = 2.5 95% CI: 1.12–4.19), polytherapy (AOR = 2.06, 95% CI: 1.18–3.37), depression (AOR = 2.37, 95% CI = 1.32–4.26), presence of comorbid diseases (AOR = 1.76, 95%CI = 1.14–3.15), and being physically active (AOR = 0.48 95% CI :0.28–0.83) were factors significantly associated with ED in hypertensive patients (Table 3).

## 4. Discussion

This study aimed to determine the prevalence of ED and associated factors among hypertensive patients who have follow-ups at governmental health institutions in Gondar city. The prevalence of ED in this study was 46.34% (95% CI: 41.6–51.1) which is similar to studies done in Egypt (43.2%) [16] and Cameroon (50.6%) [15]. However, it was lower than those reported from Thailand (56.6%) [9], Qatar (66.2%) [8], Spain (71.0%) [13], and Kenya (94.5%) [17]. This difference might be due to the sociocultural differences, the study design used, and the exclusion criteria. This finding was higher than studies from Greece (35.2%) [12], Israel (21.8%) [34], and Nigeria (41.5%) [35].

In this study, age was a significant factor for developing ED. The prevalence of ED increased as the age of study participants increased. These results are similar to those studies from Qatar [8], Thailand [9], Cameroon [15], and Nigeria [14]. Increased age is often accompanied by multiple organic diseases which can interfere with erectile function. In particular, cardiovascular and metabolic diseases with increased prevalence among older ages could play a crucial role in the pathogenesis of age-related ED [36]. Since penile erection is primarily a vascular event, it may be impaired on increasing age that causes degenerative changes in the vascular endothelium resulting in endothelial dysfunction. This in turn resulted in an alteration of vascular tone as a result of a marked reduction of the relaxation process of smooth muscle cells located in the corpus cavernosum and wall of small arteries [21]. Age-related decrease in testosterone levels could also contribute to the increased prevalence of ED [37]. Furthermore, it is recognized that there is aging-dependent decrease in the amount of functioning corporal smooth muscles. The mechanism underlying this aging-related loss of normal smooth muscle within the corporal body is believed to be mainly due to the apoptotic process primarily triggered by oxidative stress [38].

The results of this study indicated that male patients with stages I and II of hypertension exhibited ED more frequently than subjects with controlled hypertension. As the severity of hypertension increased, the prevalence of ED also increased, which is consistent with studies from Israel [34], Qatar (66.2%) [8], Greece [12], and Cameroon [15]. Our study also found that patients with hypertension follow-up for long periods were more likely to acquire ED, which is in line with studies in Thailand [9], Greece [12], and Egypt [16]. Since vascular tissue is the main contributor for penile erection, structural/functional abnormalities in penile vessels may impair the ability to achieve and maintain erection [18]. Hypertension is related to endothelial dysfunction leading to reduced nitric oxide (NO) production by the endothelium, which in turn results in ED [39]. Hypertension is also responsible for stenotic lesions secondary to atherosclerosis, smooth muscle hypertrophy of cavernosum arteries, and blood flow impairment in the penile vasculature [21].

Treatment with antihypertension medications had increased the prevalence of ED in our study participants. Patients on combination antihypertensive therapy exhibited ED more frequently (64.07%) than patients on monotherapy (25%), and the difference was statistically significant (*p* < 0.05). This finding was in line with studies in Qatar [8] and Cameroon [15], where antihypertensive polytherapy was significantly associated with ED. Combination pharmacotherapy is often prescribed in the treatment of hypertension in the majority of hypertensive patients. ED is considered a common side effect of central-acting beta-blockers and diuretics that reduce cardiac output and blood pressure, thereby decreasing blood flow to penile vasculature [40,41].

In the current study, the presence of comorbid medical illnesses such as diabetes mellitus, hypertensive heart disease, and chronic kidney disease increased the prevalence of ED. The prevalence of ED among hypertensive men with no comorbidity was 35.6%, but it was 66.9% among those with comorbidities; and the change was statistically significant which is in agreement with other studies [10,35,42]. Diabetes mellitus was the major comorbid disease found among the study participants. It affects the corpus cavernous nerve terminals and endothelial cells, resulting in a deficiency in NO synthesis and release, which in turn causes ED [43].

Moreover, this study found that ED was significantly associated with depression. This finding is supported by studies from Spain [13], China [44], and Finland [45]. Depression affects the higher centers of the brain, which initiate sexual arousal and behavior and interfere with normal erectile neurophysiology [45]. Depressed people have a low degree of sexual appetite because of their feeling of low self-esteem and guilt feeling resulting in ED [45]. However, since the study is cross-sectional, it is difficult to establish the direction of causality.

Our study also revealed that the likelihood of developing ED among physically active study participants was reduced by 48% (AOR = 0.48 95% CI: 0.28–0.83). This is in line with other studies where physical activity was statistically associated with ED [8,20,46,47]. The mechanisms by which physical activity reduces the risk of ED include improved cardiovascular fitness, endothelial function, and beneficial effects on self-esteem and mental health with a positive impact on psychological issues associated with sexual function, increase in endothelial-derived NO [48], decrease in oxidative stress [49], and increase in regenerative endothelial progenitor cells [50].

Another important finding from this study was that there were a few number of hypertensive men with ED who were approached to get counseling and medications for their sexual problems. Only 11 (2.6%) patients sought medical advice and were taking medications for treatment. This failure to seek medical attention might be due to social stigma or not considering ED as a treatable condition. As a result, ED is a common distressful problem of hypertensive patients, which is often underreported. This could further adversely affect the quality of life of the affected men.

### 4.1. Limitations of the Study

The cross-sectional nature of the study does not allow making inferences about the causal relationship. Further longitudinal studies to clarify the causal relation between ED and those predictors should be conducted. The study may have social desirability bias due to the type of data collection technique (face-to-face interviewer-administered questionnaire).

## 5. Conclusion

Nearly half of the study participants had erectile dysfunction, indicating the high burden of the problem among hypertensive patients. ED and hypertension were found to be related, as there were more cases of ED among hypertensive men. The prevalence of ED was associated with the age of patients, severity and duration of hypertension, presence of comorbidities, depression, and physical activity. Hypertensive patients with ED were not getting clinical counseling and medical treatment for their sexual problems. Therefore, assessment and management of ED should be part of routine medical care in hypertensive follow-up clinics. In addition, hypertensive patients should adequately control their blood pressure and prevent coexisting illnesses to reduce the risk of ED.

## Figures and Tables

**Table 1:** Sociodemographic characteristics of hypertensive patients attending governmental health institutions in Gondar city, Northwest Ethiopia, 2021 (*n* = 423).

Variables	Category	Frequency	Percent (%)
Age (years)	18–40	44	10.40
	41–60	201	47.52
	61–80	151	35.70
	Above 80	27	6.38

Marital status	Married	337	79.67
	Single	25	5.91
	Divorce	30	7.09
	Widowed	31	7.33

Religion	Orthodox	330	78.02
	Muslim	71	16.78
	Protestant	22	5.20

Residence	Urban	370	87.47
	Rural	53	12.53

Occupation	Government employee	109	25.77
	Merchant	91	21.51
	Nongovernment employee	80	18.68
	Farmer	53	12.53
	Daily labor	9	2.36
	Retired	81	19.15

Educational level	Cannot read and write	51	12.06
	Can read and write	71	16.78
	Primary school	93	21.99
	Secondary school	105	24.82
	Diploma and above	103	24.35

Monthly income Ethiopian Birr (ETB)	<1000	83	19.62
	1000–5000	305	72.11
	>5000	35	8.27

**Table 2:** Clinical, anthropometric, and behavioral characteristics of hypertensive patients attending governmental health institutions in Gondar city, Northwest Ethiopia, 2021 (*n* = 423).

Variables	Category	Frequency	Percent
Severity of hypertension	Controlled (<140/90)	140	33.10
	Stage I (140–159/90–99)	217	51.30
	Stage II (>160/100)	66	15.60
Duration of hypertension and its medications (year)	<5.0	228	53.9
	5.0–10.0	129	30.5
	>10.0	66	15.6
Antihypertensive medications			
Monotherapy	Diuretics	118	27.90
	Calcium channel blockers	44	10.40
	ACE inhibitor	37	8.75
Polytherapy		224	52.95
Get counseling/treatments for ED	Yes	11	2.6
	No	412	97.4
	Normal	307	72.58
	Overweight	116	27.42
Comorbidities	Yes	145	34.28
	No	278	65.72
Comorbidity type	Diabetes mellitus	60	41.38
	Cardiovascular diseases	43	29.66
	Chronic kidney disease	8	5.52
	Combinations	34	23.45
Depression status	Yes	148	34.99
	No	275	65.01
Ever alcohol drinker	Yes	320	75.65
	No	103	24.35
Current alcohol drinker	Yes	92	28.75
	No	228	71.25
Cigarette smoking	Never	351	82.98
	Former	52	12.29
	Current	20	4.73
Frequency of smoking/week	Every day	4	20
	Less than daily	16	80
Ever khat chewer	No	284	67.2
	Yes	139	32.8
Physical activity	Yes	229	54.14
	No	194	45.86

**Table 3:** Bivariable and multivariable logistic regression analysis of factors associated with erectile dysfunction among hypertensive patients attending governmental health institutions in Gondar city, Northwest Ethiopia, 2021 (*n* = 423).

Variables	Erectile dysfunction		Odds ratio (95% CI)	
	Yes (%)	No (%)	Crude	Adjusted
Age (years)				
18–40	4 (9.09)	40 (90.91)	1.00	1.00
41–60	71 (35.3)	130 (64.7)	2.58 (1.92–6.23)	2.12 (0.98–3.57)
61–80	97 (64.3)	54 (35.7)	5.45 (3.93–9.31)	3.86 (1.62–6.55)^*∗*^
>80	24 (88.9)	3 (11.1)	8.68 (4.47–12.4)	5.29 (3.38–8.07)^*∗*^

Severity of hypertension				
Controlled	24 (17.14)	116 (82.86)	1.00	1.00
Stage I	116 (53.46)	101 (46.54)	4.8 (2.92–7.90)	2.78 (1.51–5.09)^*∗*^
Stage II	56 (84.85)	10 (15.15)	6.3 (4.16–8.34)	3.54 (1.63–5.74)^*∗*^

Duration of hypertension and its medications (years)				
<5	59 (25.9)	169 (74.1)	1.00	1.00
5–10	83 (64.3)	46 (35.7)	2.1 (1.2–4.15)	1.8 (0.95–2.5)
>10	54 (81.8)	12 (18.2)	7.1 (5.6–10.7)	2.5 (1.12–4.19)^*∗*^

Antihypertensive medications				
Polytherapy	147 (65.6)	77 (34.4)	2.11 (1.35–4.78)	2.06 (1.18–3.37)^*∗*^
Monotherapy	49 (24.6)	150 (75.4)	1.00	1.00

Anti-HTN medication class				
Diuretics	25	93	2.19 (1.72–4.11)	1.27 (0.85–3.42)
Ca^2+^ CB	11	33	1.25 (1.24–3.61)	1.06 (0.71–2.18)
Diuretic + CaCB	42	47	2.12 (1.76–4.71)	1.14 (0.83–2.94)
Diuretic + BB	45	10	3.77 (1.25–5.86)	1.97 (1.26–3.75)^*∗*^
CaCB + ACEI	11	20	2.36 (1.06–5.11)	1.34 (0.76–2.77)
Diuretic + CaCB + BB	36	13	3.88 (1.87–6.20)	2.03 (1.38–3.88)^*∗*^
ACE inhibitor	13	24	1.00	1.00

Depression				
Yes	113 (76.35)	35 (23.65)	7.47 (4.72–11.80)	2.35 (1.31–4.21)^*∗*^
No	83 (30.18)	192 (69.82)	1.00	1.00

BMI				
Normal	110 (35.8)	197 (64.2)	1.00	1.00
Overweight	86 (73.15)	30 (26.85)	4.94 (3.04–8.06)	1.85 (0.98–3.5)

Physical activity				
Yes	72 (31.4)	157 (68.6)	0.25 (0.16–0.37)	0.48 (0.28–0.83)^*∗*^
No	124 (63.9)	70 (36.1)	1.00	1.00

Comorbidity				
Yes	97 (66.9)	48 (33.1)	3.6 (2.4–5.6)	1.76 (1.04–3.15)^*∗*^
No	99 (35.6)	179 (64.4)	1.00	1.00

Cigarette smoker				
Yes	28 (38.9)	44 (61.1)	1.29 (1.41–3.16)	1.15 (0.79–2.25)
No	168 (47.8)	183 (52.2)	1.00	1.00

Occupation				
Government employee	31 (28.4)	78 (71.6)	0.49 (0.12–1.97)	0.77 (0.12–5.01)
Merchant	48 (52.7)	43 (47.3)	1.39 (0.35–5.53)	0.91 (0.14–5.73)
Farmer	22 (41.5)	31 (58.5)	0.88 (0.21–3.68)	0.65 (0.09–4.33)
Nongovernment employee	41 (0.51)	39 (0.49)	1.31 (0.33–5.25)	0.89 (0.14–5.65)
Retired	50 (61.7)	31 (38.3)	2.02 (0.50–8.08)	0.39 (0.06–2.58)
Daily labor	4 (44.4)	5 (55.6)	1.00	1.00

Educational level				
Can read and write	40 (56.3)	31 (43.7)	1.57 (0.76–3.24)	2.37 (0.84–6.68)
Primary school	48 (51.6)	45 (48.4)	1.29 (0.65–2.57)	2.69 (0.91–7.99)
Secondary school	49 (46.7)	56 (53.3)	1.06 (0.54–2.08)	1.58 (0.54–4.59)
Diploma and above	36 (34.9)	67 (65.1)	0.65 (0.33–1.29)	1.89 (0.61–5.92)
Cannot read and write	23 (45.1)	28 (54.9)	1.00	1.00

CI: confidence interval; BB: beta-blocker; CaCB: calcium channel blocker; ACEI: angiotensin converting enzyme inhibitor. ^*∗*^*P* value ≤ 0.05.

## Data Availability

The research data can be obtained from authors upon reasonable request.
